# The Effects of Non-Guided Versus Guided Podcast Production on Perception of English Reading Skills in Undergraduate Nursing Students: A Quasi-Experimental Study

**DOI:** 10.3390/nursrep15120424

**Published:** 2025-11-28

**Authors:** Sirinthip Phuwayanon, Nethong Namprom, Patcharee Woragidpoonpol, Suwimol Daroonratsamee, Daniel Thomas Bressington

**Affiliations:** Faculty of Nursing, Chiang Mai University, Chiang Mai 50200, Thailand; sirinthip.p@cmu.ac.th (S.P.); patcharee.w@cmu.ac.th (P.W.); suwimol.d@cmu.ac.th (S.D.)

**Keywords:** nursing podcasts, podcast production, English reading skills, attitude, undergraduate nursing students

## Abstract

**Background:** Nurses need strong English language skills to access knowledge and promote evidence-based practice. Podcast production is a promising pedagogical strategy to improve language skills. However, the effects of podcast production on nursing students’ perceived English reading proficiency and the most effective production instructional approaches remain unreported. **Aim:** To examine the impacts of podcast production on undergraduate nursing students’ perceptions of English reading skills and compare the effectiveness of two podcast production teaching methods. **Design:** A quasi-experimental study. **Methods:** 78 third-year nursing students in Northern Thailand were divided into an experimental group (*n* = 39) and a control group (*n* = 39). The experimental group received specific guidelines for podcast production, while the control group used a non-guided method. Outcomes included students’ perceptions of their English reading skills and the experimental group’s attitudes toward podcast production guidelines. **Results:** Pre-test scores for perceived English reading skills were similar between groups (t = −1.029, *p* = 0.307). ANCOVA revealed that after controlling for pre-test scores, the control group reported significantly higher adjusted post-test scores than the experimental group (F = 5.001, *p* = 0.028). Students in the experimental group expressed positive attitudes toward the podcast production guidelines. **Conclusions:** Both podcast production approaches were effective; however, the less-guided approach showed greater improvement in students’ perceptions of their English reading skills. This approach may encourage student autonomy, creativity and deeper engagement. Podcast production emerges as a valuable student-centred learning strategy to improve perceptions of language skills, but finding a balance between support and independence during instruction seems important to maximise its potential benefits.

## 1. Introduction

The concept of learning, including learning how to be a nurse, has undergone significant changes due to the influence of various evolving factors, such as the economy, societal norms, and cultural contexts, and is shifting towards a more student-centred approach [1]. Additionally, the rapid development of technology has significantly impacted education, requiring the integration of digital education tools and technologies to enhance student engagement, motivation, and promote a more integrative and student-centred learning experience [2]. The fundamental skills, such as reading, writing, and arithmetic, form the core of student learning [3]; however, the framework of 21st-century learning skills encompasses creativity, innovation, critical thinking, problem-solving, communication, and collaboration. Creativity involves developing new ideas, methods and practices that are the basis of innovation. These competencies are essential for graduates in today’s workforce [4,5] to generate novel solutions to healthcare challenges, adapt evidence-based practices to diverse patient populations, think critically about complex clinical situations, and collaborate effectively in interprofessional teams. Higher-Order Thinking Skills (HOTS) emphasise thorough creativity, critical thinking, analysis and problem-solving. The selection of HOTS as a framework for this study is particularly relevant for nursing education. According to Bloom’s taxonomy, HOTS represent the highest levels of cognitive processes, aligning with the complex clinical reasoning and decision-making required in professional nursing practice.

In Thailand and many other countries, English is not the primary language for university students; rather, it is a second language. English proficiency is critical for nursing students, as it is the language for global communication, research, and evidence-based practices. Thai institutions providing undergraduate nursing programmes require students to take English courses as part of their curriculum. These institutions recognise English skills as an important competency in accessing current scientific knowledge, enhancing international collaboration, and delivering high-quality patient care. Consequently, our university emphasises the integration of student-centred, active learning approaches, technological proficiency, and continuous improvement of English proficiency throughout the nursing curriculum.

In 21st-century curriculum planning, student-centred and active learning approaches are essential. Nursing students must develop independent learning skills throughout their higher education. As a result, nursing faculties must design curricula incorporating student-centred learning, technology, and English language development. These components enable students to engage in self-directed learning, develop positive attitudes toward innovative learning methods [6] and improve their English language skills [7]. With the growing integration of information technology in education, students need to engage with digital tools both inside and outside the classroom. Various multimedia-based approaches have been employed, including video production, vlogging, and social media content creation. These approaches provide students with opportunities to engage in active, creative learning that improves communication and enhances language proficiency and skills related to second-language acquisition [8]. However, podcasting is a digital media tool that is becoming increasingly prevalent in higher education, particularly as a means of enhancing student participation, motivation, and facilitating knowledge acquisition [2,9]. It is typically distributed through online platforms, allowing for streaming or downloading for viewing or listening on a device [1] and offering distinct advantages over video-based methods. The audio-only format is less resource-intensive than video production, reduces performance anxiety associated with on-camera appearance, and allows students to focus specifically on audio content development and vocal delivery. Podcasting is classified as an activity designed to promote HOTS [10] as it engages multiple cognitive skills, requiring students to research information, design content, create scripts, use technology for production, and practice delivery [11], which represents the highest level of cognitive processing and promotes a more positive learning experience [10,12]. The benefits of podcasting in education contexts are well-documented, particularly in improving subject matter comprehension, English speaking and listening skills, pronunciation, and vocabulary [13] and also enhance motivation for language learning [14]. Furthermore, students enjoy and actively engage in podcast creation, appreciating its educational value [11,13].

In the context of nursing education, there have been mixed reports of the effects of podcasting on learning outcomes. For example, a study conducted in the United States revealed no significant difference in learning outcomes between nursing students in traditional lecture groups and those who learned the same content via podcasts; however, the podcast group reported satisfaction and positive learning experiences [15]. A further study demonstrated that nursing students in the United States who learned through segmented podcasts (podcasts divided into three short sections) scored higher on assessments, including multiple-choice exams and case studies, compared to those receiving traditional face-to-face lectures and unsegmented (non-stop) podcasts [16]. Whereas, an integrative review on the use of podcasts in nursing and midwifery education revealed that they resulted in improvements in learning, enhanced knowledge and skills, advanced clinical confidence, and promoted a positive perception of the technology as a learning tool [1]. Currently, podcasts are used across all educational levels to promote English language learning in Thailand, demonstrating that the approach is culturally acceptable and relevant in non-nursing educational settings. For example, a Thai study found that primary school students improved their English listening scores after learning through podcasts, with strong agreement on their benefits [17]. Another study in Thailand examining the use of podcast-based English lessons for first-year undergraduate students in a foundation English course reported significantly increased learning achievement and high student satisfaction with podcast-based lessons [18].

A key pedagogical consideration in the design of podcast-based learning activities is the extent and nature of instructional guidelines. Providing structured production guidelines can reduce cognitive load and help novice learners understand and perform complex tasks more effectively. However, it is possible that, given the highly creative nature of podcasting, the use of very structured facilitation approaches may curtail creativity, autonomy and self-directed learning as students may have less autonomy in creating and exploring different approaches [19]. The differences in instructional methods of previously reported podcast studies may provide some explanation for the discordant results. However, despite the growing use of podcasts in education, research on their application in undergraduate nurse training remains limited, and there is a lack of evidence on how using different instructional approaches may influence student nurses’ learning during podcast production. Therefore, this study aims to investigate the effects of podcasts on undergraduate nursing students’ perceptions of their English reading skills and their attitudes toward podcasts, using structured production guidelines. The findings will be helpful in curriculum development, with implications for the combination of structured instruction and student autonomy, aligning nursing education with 21st-century learning approaches.

### 1.1. Conceptual Framework

The thought process is a key component in curriculum development and management, serving as the foundation for students to develop higher-order thinking skills [20]. Critical thinking enables students to effectively solve problems and engage in lifelong learning [21]. Higher-order thinking skills emphasise thoroughly analysing and evaluating relevant information and evidence to develop well-reasoned decisions, assessments, and solutions [22,23]. Given the importance of developing nursing students’ English reading skills, this study employed podcast production as a teaching method. Learning activities in the Paediatric and Adolescent Nursing Practicum were designed to engage students in searching for and reading scientific journal articles or evidence-based practice literature on paediatric nursing. Students then summarised their findings, recorded their presentations, and published their insights as nursing podcasts. This approach aligns with Bloom’s Higher Order Thinking Skills (HOTS), which encompass designing, constructing, planning, producing, inventing, devising, and creating [10]. This course design promotes the highest level of learning, with outcomes assessed through the evaluation of nursing and midwifery content in the podcasts. The structured nature of nursing podcasts ensures their broad applicability in education. Students who successfully improve their reading and communication skills through podcast production are more likely to develop a positive attitude toward this learning method.

### 1.2. Aim

The primary aim of this study was to examine the effects of different teaching methods for podcast production on nursing students’ perceptions of their English reading skills. Additionally, a secondary aim was to explore nursing students’ attitudes towards the guidelines that prescribed a more structured approach to producing podcasts.

## 2. Materials and Methods

### 2.1. Study Design and Setting

This study utilised a quasi-experimental design to compare outcomes between two groups. Purposive sampling was used to select third-year undergraduate nursing students from the Faculty of Nursing at Chiang Mai University who were enrolled in the Paediatric and Adolescent Nursing Practicum 2 during the first semester, from 22 September to December 2021. During the course, participants practised in the Paediatric Intensive Care Unit (PICU) or the Neonatal Critical Care Unit (NCCU) for five days. Group allocation was non-random and based on students’ pre-existing class schedules. This sequential approach, with the control group participating first, was used to minimise the risk of information contamination between groups and ensure the validity of the intervention’s effects. However, the lack of randomisation in group assignment introduces potential bias. To address selection bias, the analysis further examined the pre-existing characteristics between groups to ensure comparability despite non-random assignment.

### 2.2. Participants, Inclusion and Exclusion Criteria

This study employed purposive sampling based on the available participants from the enrolled cohort (*n* = 78). This study represents an exploratory investigation of podcast production as a learning intervention in Thai paediatric nursing education. Given the lack of prior research on podcast production in nursing education and the absence of previously reported standardised effect sizes, a power analysis was not conducted. Consequently, we aimed to recruit all eligible participants for the cohort to maximise the sample size. The inclusion criteria for selecting the participants were that they were required to (1) complete the Paediatric and Adolescent Nursing course, which is a prerequisite for the Paediatric and Adolescent Nursing Practicum 2, (2) be enrolled in the regular nursing programme (Thai), and (3) be willing to participate in the study. Students who were unable to participate for the entire study period were excluded.

To minimise coercion, participants were contacted by a research assistant who was not involved in the teaching course during the recruitment process. Participants were informed and received a participant information sheet stating that participation was entirely voluntary, with written consent obtained before commencing data collection. Participants were also assured that they had the right to withdraw from this study at any time without affecting their grades.

### 2.3. Instruments

The data collection instruments were as follows:

(1) A demographic information form included sex, age, achievement scores from the podcast content, participants’ experiences and familiarity with podcasts and English language proficiency as assessed by the Common European Framework of Reference for Languages (CEFR) exam, which is an international standard for describing language ability. The English skill evaluation was based on results from the CEFR exam.
(2) A questionnaire on the perception of reading skills was developed by the researchers based on a literature review. In the context of Thailand, where English is a second language, students’ perceptions of their language abilities—particularly their confidence in English skills play a crucial role in their overall language learning success and willingness to engage with English content. Research indicates that self-confidence in language skills has a correlation with objective measures of language proficiency and students’ willingness to engage in communication [24].

This self-reported questionnaire was designed to assess students’ perceptions of their English reading skills, including reading comprehension, interpretation, and the ability to summarise meaningful content. The questionnaire consisted of 10 items, using a 5-point Likert scale that ranged from “strongly disagree” (1) to “strongly agree” (5). Higher scores indicate a higher perceived proficiency in English reading skills. Content validity was confirmed by six experts. The content validity index (CVI) for this questionnaire was 0.98, and Cronbach’s alpha score was 0.91. The full item-by-item results for this questionnaire are available in the Supplementary Material (see Table S1).

(3) A questionnaire on attitudes toward podcast production guidelines was developed by the researchers to determine the nursing students’ attitudes towards the activity. It was administered only to the experimental group as a form of process evaluation to assess their impressions of the specific educational intervention (the production guidelines). Since the control group was not exposed to these guidelines, this measure was not applicable to them. The questionnaire comprises 20 items and utilises a five-point Likert scale ranging from 1 (strongly disagree) to 5 (strongly agree), with higher scores indicating a more positive attitude. The questionnaire is divided into three subscales, asking students to reflect retrospectively on their attitude toward each phase of the podcast production process: (a) the preparation phase, (b) the production phase, and (c) the evaluation phase. Six experts reviewed this questionnaire for content validity. The content validity index (CVI) was 1.00, and Cronbach’s alpha was 0.80, indicating adequate reliability. The full item-by-item results for this questionnaire are available in the Supplementary Material (see Table S2)

### 2.4. Intervention

The intervention in this study was the provision of a specific podcast production guideline, which was provided only to the experimental group. Both the control and experimental groups participated in the same podcast production activity, which involved completing a practicum assignment, creating a podcast, and assessing perceptions of English reading skills through pre- and post-tests. The activity included summarising the article, producing a podcast, and presenting it to peers and instructors. Regarding the variability in the difficulty of English articles assigned to students, this could be a potential source of bias and confounding factors influencing the outcomes. All articles were carefully selected from peer-reviewed paediatric nursing journals to ensure consistency in academic level, similar complexity and relevance to the practicum content.

#### 2.4.1. Control Group

(1) Participants completed the demographic data form and the questionnaire on the perception of English reading skills (pre-test).
(2) Participants received non-guided teaching methods (without receiving the Guidelines for Podcast Production for Nursing Students). They were organised into small groups of three students, and each group was assigned one peer-reviewed research article on a topic in paediatric nursing. The task for each group was to summarise their assigned article, produce a podcast, and subsequently share and discuss their findings with peers and instructors.
(3) After completing the course, participants completed a questionnaire assessing their perception of English reading skills (post-test).

#### 2.4.2. Experimental Group

(1) Participants completed the demographic data form and the questionnaire on the perception of English reading skills (pre-test).
(2) Participants received guidelines for podcast production. They were organised into small groups of three students. Each group was assigned one peer-reviewed research article on a topic in paediatric nursing to use as the basis for their podcast. Subsequently, they were introduced to the detailed process of podcast production, following the specific guidelines.

The guidelines for Podcast Production are designed for Nursing Students, which outline three stages of podcast production: pre-production, production, and post-production [25,26,27]. This guideline provides guidance on the roles of both instructors and students in the podcasting process. The content for this guideline was developed by the researchers and reviewed for accuracy by six experts, including two faculty members specialising in newborn and infant nursing, two in paediatric nursing, and two representatives from the English faculty. The researchers incorporated the experts’ feedback and suggestions. Content validity testing was applied to ensure the relevance and comprehensiveness of guidelines, and the content validity index (CVI) was calculated at 1 [28].

- Pre-production stage: Each group, comprising three students, selected one research article related to paediatric nursing care observed during their practicum. Participants received guidance on selecting and summarising research articles. The instructor acted as a consultant, providing guidance on preparing a script for the podcast recording. Training was provided on podcast production techniques to optimise the final product.
- Production stage: A lecturer from the Faculty of Communication Arts provided knowledge on podcast production, including aspects of quality, length, and cover design. Each group searched for and selected one article on paediatric nursing that related to nursing care for patients in the ward where they were assigned practical training. The podcast’s content is based on the selected articles, summarising them in the students’ own words. Following this, they prepared a script, planned, and designed a presentation for their podcast, and then recorded it.
- Post-production stage: After completing their podcasts, participants uploaded their recordings to KC Moodle at Chiang Mai University, making them accessible to peers and other students. Participants also evaluated their own and their peers’ podcasts.

(3) After completing the course, participants completed questionnaires on their perceptions of English reading skills (post-test) and their attitudes toward podcast production.

In summary, both groups completed the same podcast assignment. The key difference between the control and experimental groups was that only the experimental group received guidelines for podcast production, while the control group completed the podcast task independently without guidelines.

### 2.5. Data Analysis

Descriptive analysis was conducted to summarise demographic data, perceptions of English reading skills, and nursing students’ attitudes toward podcast production, including frequencies, percentages, means and standard deviations. A dependent-sample *t*-test was employed to compare pre-test and post-test scores within both the control and experimental groups, evaluating changes in perceptions of English reading skills. An independent *t*-test was used to compare pre-test scores between groups to verify baseline equivalence.

Analysis of covariance (ANCOVA) was performed to compare post-test scores between the experimental and control groups while controlling for pre-test scores as a covariate. This approach accounts for baseline differences, increases statistical power, and reduces error variance. Before conducting ANCOVA, all assumptions were tested and verified. (1) independence of observations, ensured through separate data collection; (2) normality of residuals, assessed using the Shapiro–Wilk test, which confirmed normally distributed residuals (*p* > 0.05); (3) homogeneity of variances, verified using Levene’s test (*p* > 0.05); (4) homogeneity of regression slopes, tested by examining the interaction between pre-test scores and group, which was non-significant (*p* > 0.05); and (5) linear relationship between the covariate and dependent variable, confirmed through correlation analysis. Statistical significance was set at *p* < 0.05, as recommended in standard statistical practices for hypothesis testing [29]. All analyses were conducted using SPSS version 29.

### 2.6. Ethical Considerations

This research was approved by the Human Subjects and Ethics Committee, Faculty of Nursing, Chiang Mai University (Study Code: 2564-EXP081). The researcher’s assistant, who is neither a lecturer nor a teaching staff member, was tasked with distributing the flyer and promoting the study to eligible participants. The researchers were not involved in the recruitment process to avoid coercion and ensure that potential participants felt free from any pressure to participate. Before data collection, the researchers explained the study’s aims, details, and the educational benefits of podcast production. Participants were assured of their right to privacy and anonymity, as well as their right to withdraw from the study at any time without affecting their academic performance. The results are presented in aggregate form, with no individual identification, and are intended solely for educational purposes. To address ethical considerations regarding equal learning opportunities, it is important to clarify that both the experimental and control groups participated in the podcast production activity. The key difference in the intervention was the provision of specific “Guidelines for Podcast Production” to the experimental group, while the control group produced their podcasts without these structured guidelines. This design ensured that no students were denied the primary learning experience of creating a podcast, thereby maintaining educational equity between the groups.

## 3. Results

### 3.1. Sample Characteristics

The participants consisted of 78 nursing students, divided into a control group of 39 and an experimental group of 39. In the experimental group, the majority were female (84.6%), with an average age of 20–22 years. The average GPA was 3.09 (SD = 0.44). Most participants (59%) demonstrated A2-level English proficiency, as assessed by the CEFR exam. Regarding podcast familiarity, 61.5% were aware of podcasts, 33.3% had listened to them, but only 2.6% had experience producing podcasts. In the control group, 94.9% were female, with ages ranging from 20 to 30 years. The average GPA was 3.21 (SD = 0.43), and 48.7% of the participants demonstrated A2-level English proficiency based on the CEFR exam. Most participants (82.1%) were familiar with podcasts, 51.3% had listened to them, but none had experience producing podcasts. Statistical analysis indicated no significant differences in demographic characteristics between the control and experimental groups.

### 3.2. Scores for the Perception of English Reading Skills

According to Table 1, the experimental group’s mean pre-test score on perceptions of English reading skills was 39.97 (SD = 5.91), while the control group’s mean pre-test score was 41.31 (SD = 5.53). When comparing the perception scores for English reading skills, the difference between the control and experimental groups was not statistically significant (t = −1.029, *p* = 0.307).

An Analysis of Covariance (ANCOVA) was conducted to compare post-test scores between two groups, controlling for pre-test as a covariate. Prior to conducting the ANCOVA, all assumptions were tested and satisfied. The results indicated a significant relationship between pre-test scores and post-test scores. (F_1,75_ = 44.991, *p* < 0.001).

After adjusting for pre-test scores (evaluated at the mean of 40.64), a statistically significant difference was observed between the groups (F_1,75_ = 5.001, *p* = 0.028). The control group demonstrated significantly higher adjusted post-test mean scores (M = 44.29, SE = 0.67), compared to the experimental group (M = 42.17, SE = 0.67). The mean difference was −2.13 points (95% CI: [−4.02, −0.23], indicating that the control group scored 2.13 points higher than the experimental group with statistical significance.

Table 2 presents the within-group changes in English reading skills scores for both groups. Both groups reported a statistically significant within-group change; however, the control group demonstrated a greater within-group improvement in mean score with a significant difference (control group: t = −4.884, *p* < 0.0001; experimental group: t = −2.131, *p* = 0.040).

### 3.3. Nursing Students’ Attitude Toward Nursing Podcast Production

After completing the podcast production, students in the experimental group answered a questionnaire assessing their attitudes toward each phase of the podcast production process rather than measuring attitude changes across multiple time points. The aim was to capture students’ overall attitude across three phases of podcast production activity.

Table 3 presents the experimental group’s attitudes toward different phases of the podcast production guidelines. In the preparation phase, students reported the highest mean attitude scores (mean = 4.54, SD = 0.67), recognising the importance of utilising reputable sources and valuing the role of podcast preparation in enhancing critical and analytical thinking. During the production phase, students also expressed similarly high attitude mean scores (mean = 4.30, SD = 0.87), highlighting their appreciation of engaging language, effective presentation techniques, and the use of appealing visuals. In the evaluation phase, students’ attitudes were reported with slightly lower ratings (mean = 3.88, SD = 0.90). They strongly agreed that podcast production benefits others and is a suitable format for summarising English articles.

Students in the experimental group expressed a positive attitude towards guidelines on script preparation, emphasising flexibility in content format and the importance of introducing key topics. They acknowledged podcasting as a valuable tool for public speaking practice, but reported some anxiety about voice recording and visual presentation. Additionally, students valued peer evaluations, seeing them as opportunities for improvement, and felt podcasting boosted their confidence in applying evidence-based knowledge to nursing practice. However, some students in the experimental group had moderate concerns about the time-consuming nature of podcast production, suggesting that the process could be improved to reduce the perceived workload.

Overall, students reported favourable attitudes toward the podcast production guidelines (mean = 4.09, SD = 0.81). The slightly lower rating in the evaluation phase indicates differentiated perceptions across the different phases of podcast production. This pattern likely reflects students’ perceptions of the educational value and practical aspects of each phase, rather than showing any progression or change in attitude over the course of the study. The aim was to capture students’ overall attitude across three phases of podcast production activity (Table 3). The detailed item-by-item breakdown for all 20 attitude items is available in the Supplementary Material (Table S2).

## 4. Discussion

### 4.1. The Perception of English Reading Skills

The findings showed that both the control and experimental groups showed statistically significant improvement in their perception of English reading skills after reading English articles and creating podcasts (*p* < 0.05). This finding supports the integration of podcast production as an effective learning tool, fostering Higher-Order Thinking Skills (HOTS) in Bloom’s digital taxonomy [10]. Podcast production provided students with the opportunity to engage in higher-order cognitive tasks. These activities align closely with the principles of HOTS, as they require students to engage deeply with the content, analyse, create scripts, think critically, and apply their knowledge in a new, creative format of practice delivery. Furthermore, this outcome suggests that the use of English-language journal articles in combination for podcast production (with or without guidelines) can be beneficial for promoting perceived language development among undergraduate nursing students.

Despite both groups benefiting from the podcast production activity, the control group, which produced podcasts without structured production guidelines, showed significantly higher scores in their perception of English reading skills post-test compared to the experimental group (*p* < 0.05). Interestingly, the control group had slightly higher baseline scores; however, the difference was not statistically significant. These differences in outcomes may be attributed to the nature of reading comprehension, which involves complex cognitive interactions between textual content and the reader’s perception [30]. Additionally, reading proficiency relies on multiple factors, including grammar, vocabulary, context, and background knowledge, especially in foreign languages [31]. Therefore, while the results of our study suggest that podcast production builds active learning and may promote confidence in students’ language abilities, we did not objectively measure reading ability, and it is therefore impossible to determine if the approach sufficiently targets the specific factors required to improve reading ability performance.

In contrast with our expectations, the structured podcasting guidelines were less effective than the non-guided, more flexible approach. According to educational theory, structured guidelines can provide essential support, reduce cognitive load, ensure that learners follow the essential steps of a challenging task and avoid confusion during assignment completion [32]. However, we used Self-Determination Theory (SDT) [33] as a framework to explain the influence of autonomy on student motivation. Ryan and Deci [33] present Self-Determination Theory (SDT) as a framework that highlights the basics of psychological needs for autonomy, competence, and relatedness. Self-determination is a key factor for understanding human motivation, including intrinsic and autonomous extrinsic motivation, and well-being, all of which are directly relevant to educational contexts. Research demonstrates that intrinsic motivation and autonomous extrinsic motivation can predict positive outcomes across diverse education levels [33,34]. The highly structured guidelines may limit student autonomy, their creativity, and restrict opportunities for problem-solving and ownership of learning [35]. Conversely, fewer guidelines can promote creativity, autonomy and self-directed learning. Students may have greater autonomy in choosing and creating presentation styles, exploring different approaches and taking ownership of their learning journeys [19]. In this study, students in the control group without guidance on podcast production may have had independence in their decisions about content, organisation, and technology. This self-directed approach could have enhanced engagement with the reading material to assess relevance, structure, and presentation style, thereby strengthening reading comprehension.

Furthermore, several contextual factors may explain the observed differences related to the control group’s higher outcomes. Nursing students in the control group reported somewhat higher perceived English reading skills (pre-test) and greater prior exposure to podcasts, with a higher percentage of students reporting familiarity and listening experience than the experimental group, although these differences were not statistically significant. Additionally, the instructors for both groups played similar roles in guiding podcast production, which may have minimised the impact of instructional differences. The lack of standardised length and English difficulty level of the journal articles used in the study may have also influenced students’ perceptions of their reading skills. Differences in content difficulty, combined with individual background knowledge on the topics, could influence their self-assessment of English reading skill gains. Importantly, the experimental group had guidelines for podcast production, which provided structured step-by-step support in content preparation, scriptwriting, and technical recording for the podcast. Conversely, students in the control group produced podcasts using a non-guided approach. This was a key difference between the two groups. The findings, therefore, suggest that additional instructions via production podcast guidelines did not result in improved perceptions of their English reading skills.

Previous research has similarly reported no significant differences in correct exam responses between students learning through traditional methods and those engaged in podcast-based learning [15]. This finding aligns with research by Chol et al. [36], who found that although podcast use did not significantly enhance measured exam outcomes in economics education, students perceived podcasts to be more effective than traditional reading-based material in supporting their learning. Similarly, Yang [12] emphasised that learner-generated podcasts can support motivation and autonomy but may not necessarily result in superior language performance unless aligned with targeted skill development.

In podcast production, previous studies have shown that student-produced language learning podcasts enhance English as a Foreign Language (EFL) learners’ speaking abilities, leading to increased language confidence, fluency, and vocabulary acquisition [10]. Similarly, Taiwanese graduate students demonstrated improvements in English fluency and accuracy through podcast production, which also increased their interest in learning to speak English [37]. Additionally, Hasan and Hoon [13] found that podcast creation fosters language learning proficiency in speaking, listening, pronunciation, and vocabulary acquisition.

This study did not include an objective evaluation of the quality of the podcasts produced by each group. It is possible that the experimental group, benefiting from structured guidelines, produced higher-quality podcasts in terms of content accuracy, organisation, or language use. These factors highlight the need for further investigation into optimising podcast integration for language learning in nursing education.

In summary, learning from podcast production appears to have a positive influence on students’ perceptions of their English reading skills. However, the findings suggest that simplified instructional methods or student-directed podcast production processes may offer similar or better outcomes than structured and guided, allowing students some autonomy while saving instructional time and effort. The primary implication of this finding is that podcast-based production is effective in improving perceived English reading skills without requiring enhanced instructional scaffolding.

### 4.2. The Attitude of Nursing Students Towards Nursing Podcast Production

Our study also examined the experimental group’s attitudes towards the podcast production guidelines. Overall, this group demonstrated positive attitudes towards the guidelines and, more broadly, podcast production as a learning activity. A more nuanced analysis of the experimental group’s attitudes reveals valuable insights for educators. The highest attitude scores were observed during the preparation phase (mean = 4.54), suggesting that students were highly motivated and engaged when planning and researching their topics with the support of the guidelines. This initial structure may have reduced anxiety and built confidence. Attitudes remained very positive during the production phase (mean = 4.30), though slightly lower, perhaps reflecting the technical challenges and time commitment involved in recording and editing. The lowest (though still positive) attitude was toward the evaluation phase (mean = 3.88). This finding is particularly interesting, as it may indicate that processes involving self-assessment and peer evaluation, while valuable, can also be a source of stress or anxiety for students. They acknowledged podcasting as a valuable tool for public speaking practice but reported some anxiety about voice recording and visual presentation (see Table S2 in Supplementary Material). This detailed breakdown provides more than a simple measure of satisfaction; it offers a roadmap for educators, highlighting that while students embrace the creative aspects of podcasting, specific support may be needed to navigate the technical and evaluative stages of such projects. This result aligns with previous research conducted by Armstrong et al. [38], which has shown that students experience enjoyment in podcast production and develop positive attitudes toward learning through this medium. Consistently, research has indicated that students generally have positive attitudes and perceived benefits in creating podcasts [37]. Phillips [11] also noted that students who create podcasts improve their language confidence, fluency, and understanding, which makes it a valuable tool for self-directed learning. Additionally, these findings corroborate prior research suggesting that participants expressed positive attitudes toward creating English podcasts, enjoying and recognising the positive impact of this activity on their English-speaking confidence [12].

In this study, positive attitudes were associated with perceptions of English language development. The podcasting assignment required students to read academic nursing articles, summarise, and transfer the content into podcast scripts. From the perspective of English proficiency, this process facilitated students’ practice of vocabulary, paraphrasing and grammar application [13] while also enhancing their understanding of nursing content. Additionally, the task’s requirement to convey complex ideas in accessible English requires careful attention to language accuracy and clarity, both of which contribute to proficiency.

However, despite the positive attitudes, the findings of this study indicate that the control group, who produced podcasts without structured guidelines, achieved significantly higher post-test scores in their perception of English reading skills than the experimental group who received structured guidance in podcast production (*p* < 0.05). Nevertheless, the strong positive response to podcast production emphasises its pedagogical value, students were highly motivated, actively engaged, and demonstrated creativity and autonomy which is an important educational outcome in itself.

### 4.3. Implications for Nursing Education

The findings in this study indicate that incorporating learner-generated podcasts as a student-centred learning strategy within nursing curricula results in improvements in nursing students’ perceptions of their English language reading skills. The integration of reading academic English articles with podcast creation is likely to help improve English language skills, bolster higher-order thinking, and encourage self-directed learning in nursing students, particularly in contexts where English is not the primary language. Furthermore, the process of podcast production develops critical thinking, creativity, technology-enhanced learning and digital literacy. By developing English proficiency and actively engaging, students can access global nursing knowledge and synthesise and apply theoretical knowledge to clinical practice.

### 4.4. Study Limitations

This study presents several limitations. The sample size was based on the number of available participants from the enrolled cohort and their willingness to participate, rather than an a priori power analysis, which may have impacted the statistical power of the study. Future studies should incorporate power analysis to ensure sufficient sample sizes and more robust statistical outcomes. The small sample size of participants collected from a single institution may restrict the generalisability of the findings. Additionally, the generalisability to other educational settings may be limited as this study was conducted within the Thai cultural context and used Thai-language outcome measures to ensure accurate comprehension. Future research should involve larger and more diverse participants to improve external validity and enhance generalisability across diverse educational settings. Additionally, variations in participants’ prior exposure to podcasts and differences in their baseline English proficiency levels were not controlled, which may have influenced the results. These variations are likely to have arisen from the non-random allocation of students into groups. This study also did not standardise the length or complexity of the reading materials, which may have affected students’ perceptions of their English reading skills. Furthermore, the study relied on self-reported measures to assess English reading skill perceptions and attitudes toward podcast production, which may introduce response bias. An additional limitation is the potential Hawthorne effect, as students in the experimental group may behave or perform differently, influenced by their awareness of participating in being observed in an innovative study setting [39]. This motivation to pay attention and perform well could have inflated positive outcomes and introduced bias, making it difficult to determine the extent of their improvements. Future studies should consider implementing strategies to minimise this effect and incorporate objective assessments, such as standardised reading comprehension tests and qualitative evaluations of podcast content, to provide a more comprehensive understanding of learning outcomes.

### 4.5. Suggestions for Future Research

Future research should explore larger and more varied samples to improve the generalisability of findings and to examine the consistency of observed effects across different nursing education contexts. Standardisation of reading materials and controlling prior podcast exposure of participants can provide clearer insights into the effectiveness of podcast production. The integration of structured reading strategies with podcast production may optimise language learning outcomes. The standardised reading tests and qualitative evaluations of the podcast could strengthen the findings. In addition, interviews or focus groups from qualitative approaches could provide a deeper understanding of students’ experiences, challenges, and perceived facilitators of the learning process through podcast production. Longitudinal studies research should be conducted to assess the effects of learner-generated podcasts on nursing students’ English language skills, professional communication proficiency, and interprofessional collaboration. Finally, exploring the application of podcast creation in other nursing departments and across health professions education could help establish its broader use as an innovative tool of learning and a student-centred teaching strategy.

## 5. Conclusions

While podcast production was effective for both groups, the less-guided approach (control group) resulted in a significantly greater improvement in students’ perceptions of their English reading skills compared to the highly structured guideline approach (experimental group). Within creative, student-centred tasks like podcast production, providing excessive structure in guidelines may not always yield the best outcomes for perceived skill development. A less guided approach may develop greater student autonomy, creativity, and deeper engagement with the source material as students problem-solve independently. This suggests that the optimal instructional strategy may involve balancing foundational support with the freedom for students to take ownership of their learning process. Future pedagogical designs should consider how to support, rather than over-prescribe, such creative learning activities.

## Figures and Tables

**Table 1:** Comparison of perceived English reading skills between the experimental and control groups using ANCOVA (*n* = 78).

	Experimental Group (*n* = 39)	Control Group (*n* = 39)	Test Statistic	*p*-Value	Effect Size (ηp^2^)
Pre-test					
Mean (SD)	39.97 (5.91)	41.31 (5.53)	t = −1.029	0.307	
Post-test					
Observed Mean (SD)	41.79 (5.62)	44.67 (4.82)	t = −2.422	0.018 *	
Adjusted Mean (SE) ^a^	42.17 (0.67)	44.29 (0.67)	F = 5.001	0.028 *	η^2^ = 0.063
95% CI for Adj. Mean	[40.84, 43.50]	[42.96, 45.63]			

Note: * *p* < 0.05, ^a^ Adjusted means, and standard errors (SE) estimated from ANCOVA with pre-test scores as the covariate (evaluated at covariate mean = 40.64). Mean difference (adjusted) = −2.13, 95% CI [−4.02, −0.23] (Experimental-Control).

**Table 2:** Within-group comparison of English reading skills pre- and post-test of the control and experimental group, using a dependent *t*-test (*n* = 78).

	Mean	SD	t	*p*-Value
Control group (*n* = 39)				
Pre-test	41.31	5.53	−4.884	<0.001 **
Post-test	44.67	4.83		
Experimental group (*n* = 39)				
Pre-test	39.97	5.91	−2.131	0.040 *
Post-test	41.79	5.62		

Note * *p* < 0.05, ** *p* < 0.01.

**Table 3:** Mean scores and standard deviation of the attitude of nursing students in the experimental group toward podcast production guidelines (*n* = 39).

Phase	Mean	SD
Preparation phase	4.54	0.67
Production phase	4.30	0.87
Evaluation phase	3.88	0.90
Total	4.09	0.81

Note: the phases listed are derived from the attitudes toward the podcast production guidelines questionnaire instrument.

## Data Availability

The data used in this study can be requested from the author of the manuscript.

## Supplementary Materials

The following supporting information can be downloaded at: https://www.mdpi.com/article/10.3390/nursrep15120424/s1, Table S1: Between-group comparison of perception of English reading skills scores pre-test. Table S2: Mean scores and standard deviation of the attitude of nursing students in the experimental group toward podcast production guidelines (*n*=39).

## Public Involvement Statement

No public involvement in any aspect of this research.

## Guidelines and Standards Statement

This manuscript was drafted against the Strengthening the Reporting of Observational Studies in Epidemiology (STROBE) guidelines.

## Abbreviations

The following abbreviations are used in this manuscript:

EFL	English as a Foreign Language
HOTS	Higher Order Thinking Skills
PICU	Paediatric Intensive Care Unit
NCCU	Neonatal Critical Care Unit
