# Supplementary material for: The Effects of Non-Guided Versus Guided Podcast Production on Perception of English Reading Skills in Undergraduate Nursing Students: A Quasi-Experimental Study
Source: Nurs Rep. 2025 Nov 28;15(12):424. doi: 10.3390/nursrep15120424 (PMC12735567; doi:10.3390/nursrep15120424)
Supplement: Supplementary file 1 [file nursrep-15-00424-s001.zip › nursrep-3914741-supplementary.pdf]

Table S1-1: Between-group comparison of perception of English reading skills scores **pre-test**

**Items**	**Pre-test** (M ± SD)		**p-value**
	Control group (n=39)	Experimental group (n=39)	
1. Creating a nursing podcast enhances my ability to understand nursing content in English articles I read.	4.21 ± 0.62	3.87 ± 0.66	0.027[a*]
2. Creating a nursing podcast helped me learn more English vocabulary.	4.46 ± 0.56	4.49 ± 0.64	0.675[a]
3. Creating a nursing podcast has improved my understanding of the skills needed to translate English articles.	4.26 ± 0.75	4.21 ± 0.83	0.842[a]
4. The use of translation programs, such as Google Translate, serves as a helpful tool for translating English articles.	3.95 ± 0.89	3.79 ± 1.01	0.618[a]
5. Creating a nursing podcast helped me understand how to capture the main idea of each paragraph in an English article.	4.21 ± 0.80	4.00 ± 0.76	0.203[a]
6. Creating a nursing podcast allowed me to summarise the content derived from reading an English article.	4.21 ± 0.80	4.00 ± 0.80	0.226[a]
7. Creating a nursing podcast has increased my interest and commitment to reading and summarising English articles.	4.08 ± 0.81	3.79 ± 0.80	0.086[a]
8. Creating a nursing podcast helped me develop motivation to read and summarise English articles.	3.74 ± 0.85	3.72 ± 0.89	0.861[a]
9. Creating a nursing podcast has prompted me to analyse the content of English articles critically in order to creatively transform them into a podcast.	4.23 ± 0.78	4.10 ± 0.72	0.357[a]
10. Creating a nursing podcast is an appropriate method for developing nursing students' English reading skills.	3.97 ± 0.74	4.00 ± 0.73	0.956[a]
Total score	41.31 ± 5.53	39.97 ± 5.91	0.307[b]

Note * $p < 0.05$, a = Mann-Whitney U test, b = independent t-test

Table S1-2: Between-group comparison of perception of English reading skills scores **post-test.**

Items	**Post-test** (M ± SD)		p-value
	Control group (n=39)	Experimental group (n=39)	
1. Creating a nursing podcast enhances my ability to understand nursing content in English articles I read.	4.51 ± 0.51	4.41 ± 0.64	0.586[a]
2. Creating a nursing podcast helped me learn more English vocabulary.	4.59 ± 0.68	4.56 ± 0.60	0.669[a]
3. Creating a nursing podcast has improved my understanding of the skills needed to translate English articles.	4.54 ± 0.56	4.36 ± 0.71	0.307[a]
4. The use of translation programs, such as Google Translate, serves as a helpful tool for translating English articles.	4.21 ± 0.86	3.79 ± 1.01	0.070[a]
5. Creating a nursing podcast helped me understand how to capture the main idea of each paragraph in an English article.	4.41 ± 0.60	4.33 ± 0.70	0.737[a]
6. Creating a nursing podcast allowed me to summarise the content derived from reading an English article.	4.51 ± 0.56	4.28 ± 0.69	0.135[a]
7. Creating a nursing podcast has increased my interest and commitment to reading and summarising English articles.	4.38 ± 0.63	3.87 ± 0.86	0.007[a]**
8. Creating a nursing podcast helped me develop motivation to read and summarise English articles.	4.33 ± 0.81	3.87 ± 0.86	0.014[a]*
9. Creating a nursing podcast has prompted me to analyse the content of English articles critically in order to creatively transform them into a podcast.	4.59 ± 0.64	4.31 ± 0.77	0.072[a]
10. Creating a nursing podcast is an appropriate method for developing nursing students' English reading skills.	4.59 ± 0.64	4.00 ± 0.92	0.001[a]**
**Total score**	44.67 ± 4.82	41.79 ± 5.62	.018[b]*

Note * $p < 0.05$, ** $p < 0.01$, a = Mann-Whitney U test, b = independent t-test

Table S1-3: Within-group comparison of English reading skills pre- and post-test of the **control group.**

**Items**	**Control group** (n=39)		**p-value**
	Pre-test (M ± SD)	Post-test (M ± SD)	
1. Creating a nursing podcast enhances my ability to understand nursing content in English articles I read.	4.21 ± 0.62	4.51 ± 0.51	0.005[a]**
2. Creating a nursing podcast helped me learn more English vocabulary.	4.46 ± 0.56	4.59 ± 0.68	0.096[a]
3. Creating a nursing podcast has improved my understanding of the skills needed to translate English articles.	4.26 ± 0.75	4.54 ± 0.56	0.008[a]**
4. The use of translation programs, such as Google Translate, serves as a helpful tool for translating English articles.	3.95 ± 0.89	4.21 ± 0.86	0.141[a]
5. Creating a nursing podcast helped me understand how to capture the main idea of each paragraph in an English article.	4.21 ± 0.80	4.41 ± 0.60	0.059[a]
6. Creating a nursing podcast allowed me to summarise the content derived from reading an English article.	4.21 ± 0.80	4.51 ± 0.56	0.014[a]*
7. Creating a nursing podcast has increased my interest and commitment to reading and summarising English articles.	4.08 ± 0.81	4.38 ± 0.63	0.018[a]*
8. Creating a nursing podcast helped me develop motivation to read and summarise English articles.	3.74 ± 0.85	4.33 ± 0.81	<0.001[a]**
9. Creating a nursing podcast has prompted me to analyse the content of English articles critically in order to creatively transform them into a podcast.	4.23 ± 0.78	4.59 ± 0.64	0.006[a]**
10. Creating a nursing podcast is an appropriate method for developing nursing students' English reading skills.	3.97 ± 0.74	4.59 ± 0.64	<0.001[a]**
**Total score**	41.31 ± 5.53	44.67 ± 4.82	<0.001[b]**

Note * $p < 0.05$, ** $p < 0.01$, a = Wilcoxon signed-rank test, b = dependent t-test

Table S1-4: Within-group comparison of English reading skills pre- and post-test of the **experimental group**

Items	**Experimental group** (n=39)		**p-value**
	Pre-test (M ± SD)	Post-test (M ± SD)	
1. Creating a nursing podcast enhances my ability to understand nursing content in English articles I read.	3.87 ± 0.66	4.41 ± 0.64	<0.001[a]**
2. Creating a nursing podcast helped me learn more English vocabulary.	4.49 ± 0.64	4.56 ± 0.60	0.405[a]
3. Creating a nursing podcast has improved my understanding of the skills needed to translate English articles.	4.21 ± 0.83	4.36 ± 0.71	0.257[a]
4. The use of translation programs, such as Google Translate, serves as a helpful tool for translating English articles.	3.79 ± 1.01	3.79 ± 1.01	0.945[a]
5. Creating a nursing podcast helped me understand how to capture the main idea of each paragraph in an English article.	4.00 ± 0.76	4.33 ± 0.70	0.014[a]*
6. Creating a nursing podcast allowed me to summarise the content derived from reading an English article.	4.00 ± 0.80	4.28 ± 0.69	0.022[a]*
7. Creating a nursing podcast has increased my interest and commitment to reading and summarising English articles.	3.79 ± 0.80	3.87 ± 0.86	0.681[a]
8. Creating a nursing podcast helped me develop motivation to read and summarise English articles.	3.72 ± 0.89	3.87 ± 0.86	0.225[a]
9. Creating a nursing podcast has prompted me to analyse the content of English articles critically in order to creatively transform them into a podcast.	4.10 ± 0.72	4.31 ± 0.77	0.059[a]
10. Creating a nursing podcast is an appropriate method for developing nursing students' English reading skills.	4.00 ± 0.73	4.00 ± 0.92	0.684[a]
Total score	39.97 ± 5.91	41.79 ± 5.62	0.040[b]*

Note * $p < 0.05$, ** $p < 0.01$, a = Wilcoxon signed-rank test, b = dependent t-test

Nursing students' attitude toward nursing podcast production

Table S2: Mean scores and standard deviation of the attitude of nursing students in the experimental group toward podcast production guidelines (n=39).

Items	Mean	SD	Attitude level
**Preparation Phase**			
1. The creation of nursing podcasts requires selecting research articles or academic content from credible sources.	4.79	0.52	very good
2. Reading to summarise content prior prepare the script for recording promotes critical and analytical thinking, which is more effective than simply reading it.	4.67	0.62	very good
3. The preparation of the podcast script and the arrangement of the content should not be structured but should specify key topics that need to be presented.	4.15	0.87	good
**total**	**4.54**	**0.67**	**very good**
**Production phase**			
4. Recording an audio for the podcasts provides an opportunity to practice public speaking.	4.15	0.81	good
5. Recording a nursing podcast using good content through engaging language and an interesting speaking style is important for attracting listeners.	4.59	0.64	very good
6. The voice recording in a podcast should not be time limited.	4.00	1.10	good
7. The cover image or symbol of the podcast plays a crucial role in attracting the audience's attention.	4.56	0.79	very good
8. I am anxious about the images and voice recording during the nursing podcast production.	4.18	0.99	good
**total**	**4.30**	**0.87**	**very good**
**Evaluation Phase**			
9. I want to share my nursing podcast with students in the other groups.	3.74	0.99	good
10. I want to get an evaluation of my podcasts from my peers in different groups.	3.59	1.02	good
11. Receiving feedback from my peers or listeners encourages me to improve my nursing podcast.	4.18	0.82	good
12. I enjoy producing nursing podcasts.	3.46	1.05	good
13. I am satisfied with my nursing podcasts.	4.13	0.83	good

Items	Mean	SD	Attitude level
14. To have a hand in nursing podcast production is beneficial to me.	4.28	0.69	very good
15. Nursing podcast production can benefit others.	4.41	0.59	very good
16. Creating nursing podcasts is a suitable format for presenting summaries of English articles.	4.21	0.86	very good
17. The process of nursing podcast production has reinforced my confidence in utilising new evidence-based knowledge in nursing practice.	4.18	0.72	good
18. The process of nursing podcast production wastes a lot of my time.	3.28	1.21	moderate
19. The process of nursing podcast production has been complicated for me.	3.38	1.16	moderate
20. Nursing podcast production is an appropriate and effective learning activity for nursing students.	3.82	0.97	good
**total**	**3.88**	**0.90**	**good**
**Total**	4.09	0.81	good
